# Levels of Angiotensin-Converting Enzyme and Apolipoproteins Are Associated with Alzheimer’s Disease and Cardiovascular Diseases

**DOI:** 10.3390/cells11010029

**Published:** 2021-12-23

**Authors:** Chun Xu, Debra Garcia, Yongke Lu, Kaysie Ozuna, Donald A. Adjeroh, Kesheng Wang, on behalf of the Alzheimer’s Disease Neuroimaging Initiative

**Affiliations:** 1 Department of Health and Biomedical Sciences, College of Health Professions, University of Teas Rio Grande Valley, Brownsville, TX 78520, USA; debra.garcia03@utrgv.edu (D.G.); kaysie.ozuna01@utrgv.edu (K.O.); 2 Department of Biomedical Sciences, Joan C. Edwards School of Medicine, Marshall University, Huntington, WV 25755, USA; luy@marshall.edu; 3 Lane Department of Computer Science & Electrical Engineering, West Virginia University, Morgantown, WV 26506, USA; don@csee.wvu.edu; 4 Department of Family and Community Health, School of Nursing, West Virginia University, Morgantown, WV 26506, USA

**Keywords:** Alzheimer’s disease, cardiovascular diseases, angiotensin-converting enzyme, apolipoproteins, ApoB, ApoH

## Abstract

Angiotensin-converting enzyme-1 (ACE1) and apolipoproteins (APOs) may play important roles in the development of Alzheimer’s disease (AD) and cardiovascular diseases (CVDs). This study aimed to examine the associations of AD, CVD, and endocrine-metabolic diseases (EMDs) with the levels of ACE1 and 9 APO proteins (ApoAI, ApoAII, ApoAIV, ApoB, ApoCI, ApoCIII, ApoD, ApoE, and ApoH). Non-Hispanic white individuals including 109 patients with AD, 356 mild cognitive impairment (MCI), 373 CVD, 198 EMD and controls were selected from the Alzheimer’s Disease Neuroimaging Initiative (ADNI) dataset. Multivariable general linear model (GLM) was used to examine the associations. *ApoE* ε4 allele was associated with AD, as well as ApoAIV, ApoB and ApoE proteins, but not associated with CVD and EMD. Both AD and CVD were associated with levels of ACE1, ApoB, and ApoH proteins. AD, MCI and EMD were associated with levels of ACE1, ApoAII, and ApoE proteins. This is the first study to report associations of ACE1 and several APO proteins with AD, MCI, CVD and EMD, respectively, including upregulated and downregulated protein levels. In conclusion, as specific or shared biomarkers, the levels of ACE1 and APO proteins are implicated for AD, CVD, EMD and *ApoE* ε4 allele. Further studies are required for validation to establish reliable biomarkers for these health conditions.

## 1. Introduction

Alzheimer’s disease (AD) is a progressive disease that affects memory through the degeneration of brain cell connections and the cells themselves [1]. The pathogenesis of AD is associated with abnormal lipid metabolism [2]. Lipids are transported by binding to apolipoproteins. Abnormal aplipoprotein metabolism may lead to an increased clearance by macrophages and result in atherosclerosis. Among the pool of apolipoproteins, apolipoprotein E (ApoE) is well studied, and ApoE is found in plasma as well as in cerebrospinal fluid (CSF). The human *ApoE* gene is functionally polymorphic and consists of three allele variants—ε2, ε3, and ε4 [3]. The *ApoE* ε3 allele is the most common allele, followed by ε4 allele [2]. Due to its distinct role in lipid metabolism, *ApoE* variants naturally influence cholesterol transport and homeostasis. The relation between *ApoE* ε4 and CVD might also determine the risk and prevalence of dementia [4]. ApoE absence results in a spike in the plasma cholesterol levels, and the start of atherosclerosis [5]. Indeed, over 60% of people with AD have at least one *ApoE* ε4 allele [6]. ApoE is detected in the brain [7] and is synthesized at several tissues, and secreted in a variety of cells, and because of its avidity for lipids, it can be secreted in a lipid-poor form [8]. Functions of ApoE involve cholesterol efflux, more specifically involved in cholesterol-loaded macrophage foam cells, and other atherosclerosis-relevant cells [9]. Therefore, *ApoE* gene is a potential candidate gene, not only for AD, but also for CVD. However, there is limited study of *ApoE* genetic variants in association with the level of other apolipoproteins (APOs).

Increased studies suggested shared risk factors, pathophysiology and clinical presentations among three common diseases, AD, CVD and endocrine-metabolic diseases (EMD) [10]. Susceptibility to metabolic disturbances and the response to disease are determined by the variations in the genes that code for APOs, including their receptors and their interaction with the environment [11]. CVD is characterized by vessels and atherosclerosis. In CVD, certain *ApoE* genotypes are responsible for initiating the synthesis in microphages that also cause the formation of high density-like lipoproteins that affect the cholesterol transport to the liver in reverse [12]. Receptors on apolipoproteins serve not only as risk factors for CVD and causes for degeneration of the central nervous system (CNS), but also serve to control lipid metabolism [13], including EMDs, which have been associated with immune system imbalance and in endothelial dysfunction that leads to the impaired control of constricted blood vessels and contributes to the pathogenesis of both CVD and EMD [14]. It is well established that the ApoE protein is associated with processes of lipid metabolism, possibly involved several diseases [8].

Various studies show shared physiological, pathology and clinical manifestations among AD, CVD, and EMD, while risk factors associated with CVD and a cluster of EMD significantly influence the incidence of AD. For example, imbalanced blood cholesterol levels linked to metabolic disorders have been associated with the risk for AD [15]. Furthermore, the *ApoE* ε4 allele is associated with AD, so cholesterol association with APOs is correlated. Genes, environmental factors, and their complex interactions contribute to the development of chronic, multi-factorial health conditions such as AD, CVD, or EMD. Most importantly, studies report shared *ApoE* ε4 allele among several chronic health conditions, including AD, CVD, and metabolic phenotypes [16]. *ApoE* ε4 allele increases the risk of coronary heart disease by approximately 40% [17]. The result of a stimulation between gene-environment interaction would be minor metabolic abnormalities in AD that could only be measured through a given period [18].

There are limited studies in the associations of biomarkers (e.g., APO protein levels) with AD, CVD, EMD and *ApoE* gene [19]. The pathogenesis of AD and CVD involves the accumulation of an amyloid-beta (Aβ) peptides [20]. Angiotensin-Converting Enzyme-1 (ACE1) has been suggested to play a direct role in regulating the degradation of Aβ, a recent cross-sectional regression analysis concluded that ACE protein level and CSF activity were significantly lower in subjects with AD therefore strengthening the hypothesis that ACE degrades Aβ [21]. The deposition of Aβ causes tissue inflammation and organ dysfunction, including the brain and the heart [22]. The results of a recent study suggest that subclinical cardiac involvement in AD is likely associated with Aβ [23]. One connection between *ApoE* gene and Aβ is that ApoE is a factor that influences the outcome of Aβs latency in the extracellular space [24]. Since ApoE4 is the most unstable member of the three isoforms, it is known to have poor cholesterol delivery to neurons and is involved in the accumulation of Aβ aggregation. A more detailed analysis of this interaction would show that ApoE binds to at least 3 of the receptors in the CNS. The receptors that can result in the attachment with ApoE protein would be the low-density lipoprotein receptor (LDL-R), very low-density lipoprotein receptor (VLDL-R), and low-density lipoprotein like receptor (LRP) [25]. An earlier study showed that the *ApoE* ε4 allele is associated with coronary atherosclerosis in AD patients due to the emergence of the angiotensin-converting enzyme DD genotype (*ACE*-DD), which is increased as the patient ages [26].

Another key biomarker is ACE enzyme. The *ACE* gene has become one of the most researched candidate genes for its influence on the risk for hypertension, along with cardiovascular diseases [27]. Moreover, ACE is also responsible for converting angiotensin I to angiotensin II, and increased levels of angiotensin II cerebrovascular constriction and damage lead to an increased risk of AD [28]. Moreover, APOs also show association with AD, CVD, and EMD [29]. More specifically, apolipoprotein B (Apo-B) may be involved in the pathophysiology of AD and CVD; however, there is limited study on this potential involvement [30]. Atherosclerosis is known to be the most common contributor to CVD, and all the atherogenic lipoproteins have a single Apo-B molecule as their structural protein [31]. It has been shown that serum Apo-B, as one biomarker, is a strong predictor of CVD [32] and could play a role in regulating physical function in addition to indirectly impacting the lipid profile [30].

Given the potential links of these protein levels with the diseases discussed above, we hypothesized that levels of ACE1 enzyme and APO proteins, as potential biomarkers may be involved in the developments of AD, CVD and EMD via abnormal cholesterol efflux, cholesterol transport, immune system imbalance and/or in endothelial dysfunction. However, there has been limited, at times contradictory, studies on the interplay between *ApoE* alleles and the levels of these ten proteins (one ACE1, nine APOs; namely, ApoAI, ApoAII, ApoAIV, ApoB, ApoCI, ApoCIII, ApoD, ApoE, and ApoH) with AD, CVD, and EMD. Therefore, the aim of the present study is to examine the associations of *ApoE* gene, AD, CVD and EMD with the levels of ACE1 and the nine APOs.

## 2. Materials and methods

### 2.1. Study Subjects

Data used in the preparation of this article were obtained from the Alzheimer’s Disease Neuroimaging Initiative (ADNI) database (adni.loni.usc.edu, accessed on 17 May 2021). The ADNI was launched in 2003 as a public-private partnership. The primary goal of ADNI has been to test whether serial magnetic resonance imaging (MRI), positron emission tomography (PET), other biological markers, and clinical and neuropsychological assessments can be combined to measure the progression of mild cognitive impairment (MCI) and early AD. The ADNI study began in 2004 as a multicenter that provides services to the United States and Canada. The ADNI is an ongoing, longitudinal, multicenter study designed to develop clinical, imaging, genetic, and biochemical biomarkers for the early detection and tracking of AD. For this study, the merged data were used from several components of ADNI. There was an Institutional Review Board exemption for current study due to secondary data analysis.

### 2.2. Measures

Demographic variables included age, gender, and educational levels. Gender was self-reported as either male or female. Age and education were continuous variables in years. All individuals in the present study are non-Hispanic white. AD was diagnosed using NINCDS/ADRDA criteria for probable AD [33]. There were 109 individuals with AD, 356 with MCI, and 53 with cognitive normal (CN) as control subjects. CVD and EMD were from data (MedHist) in ADNI. Diagnoses for 373 CVD and 198 EMD were based on self-reported on CVD and EMD were defined as Yes (with CVD or EMD disease histories, respectively) or No (without these two disease histories, respectively) (Table 1).

The data of *ApoE* genotypes were extracted from the ADNI database. *ApoE ε4* genotyping was performed on DNA samples obtained from subjects’ blood, using an ApoE genotyping kit. The two SNPs characterizing *ApoE* ε2/ε3/ε4 status (rs429358 and rs7412) were genotyped separately and merged with the array data sets. *ApoE ε4* carriers were defined as individuals with at least one ε4 allele (ε4/ ε4 designated as *ApoE-ε4*-2, ε4/ ε3 or ε4/ ε2 as *ApoE-ε4*-1+), while non-carriers were defined as individuals with no ε4 allele (*ApoE ε4*-0) (Table 1).

The data on ACE1 and APOs were from a subset of “Biomarkers Consortium Plasma Proteomics Project RBM multiplex data” in ADNI. In the present study, we selected 10 proteins, namely, ACE1 and 9 APO proteins (ApoAI, ApoAII, ApoAIV, ApoB, ApoCI, ApoCIII, ApoD, ApoE, and ApoH). After merging demographic variables, AD diagnosis, medical history variables, *ApoE* genotypes and protein data, the total sample size is 518 (Table 1), including 109 with AD, 356 with MCI, and 53 with CN (cognitive normal). There are a total of 373 patients with CVD, 145 subjects without CVD (called non-CVD), as well as 198 patients with EMD and 320 subjects without EMD (called non-EMD).

### 2.3. Statistical Methods

The categorical variables were presented in their raw values along with the proportions for categorical variables. Continuous variables such as age and education are presented in the form of mean ± SD. Chi-square test was used to examine the associations of categorical variables with AD diagnosis, CVD, and EMD. One-way ANOVA was performed to determine differences in continuous variables among AD diagnosis, CVD, and EMD status.

**Figure 1:**
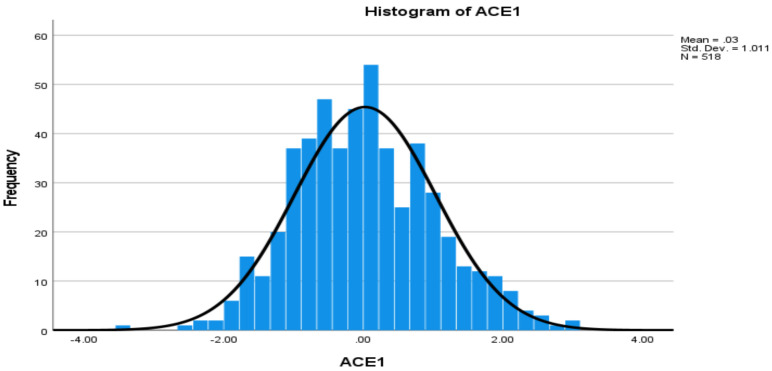
Histogram of angiotensin-converting enzyme 1 (ACE1) with normal curve, based on Z score.

**Figure 2:**
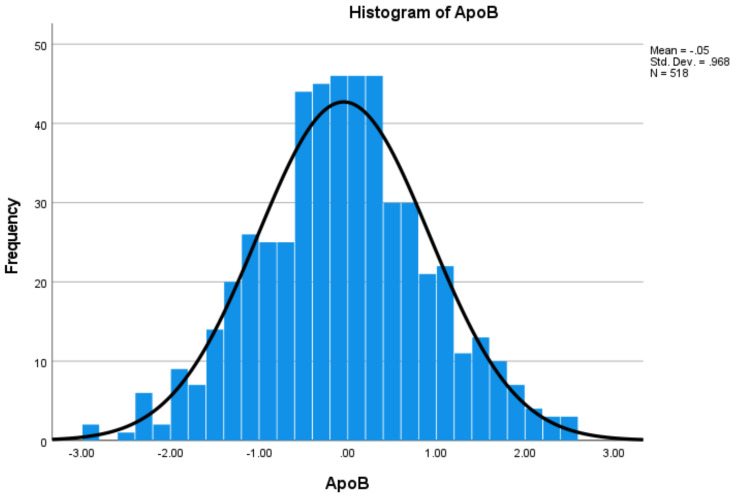
Histogram of ApoB with normal curve based on Z score.

The distributions of protein levels may be skewed. The Z score was computed for each protein using the mean and standard deviation. Values with a Z score greater than 3.5 or less than −3.5 were considered as possible outliers [34]. After removing outlier individuals, respective skewness and kurtosis values for each of the 10 proteins were within (−2, 2), indicating closeness to normal distributions. The histograms of Z scores for two proteins are presented in Figure 1 and Figure 2, respectively.

Pearson and Spearman correlations were computed to test associations among 10 proteins using the Z scores. To further analyze the relationships among the ten proteins, the VARCLUS procedure in SAS 9.4 (SAS Institute, Cary, CA, USA) was used to explore the potential clusters of variables with strong correlations with each other within a cluster, and weak correlations, with variables in other clusters [35].

Independent samples *t* test was used to compare the means in each of the ten proteins between two groups with respect to AD vs. CN, MCI vs. CN, CVD vs. non-CVD, EMD vs. non-EMD, and *ApoE* ε4-1+ vs. *ApoE* ε4-0. Then, the multivariable general linear model (GLM) was used to examine the associations of *ApoE* genotypes, AD, CVD, and EMD with each of the 10 proteins, adjusted for age, gender and education. All statistical analyses were performed using SAS (version 9.4).

## 3. Results

### 3.1. Descriptive Statistics

Age, gender, and education level were not associated with AD diagnosis (*p* = 0.9176, 0.1449 and 0.2134, respectively), however, a significant association was observed of *ApoE* ε4 allele with AD diagnosis (*p* < 0.0001) (Table 1). There were no significant associations between CVD diagnostic status and age, education level, and *ApoE* ε4 allele, but a significant difference was observed between gender and CVD diagnosis (*p* = 0.0306). There was no association of EMD diagnosis with age, gender, education, and *ApoE* ε4 allele.

### 3.2. Correlation Analysis and Variable Cluster Analysis

**Figure 3:**
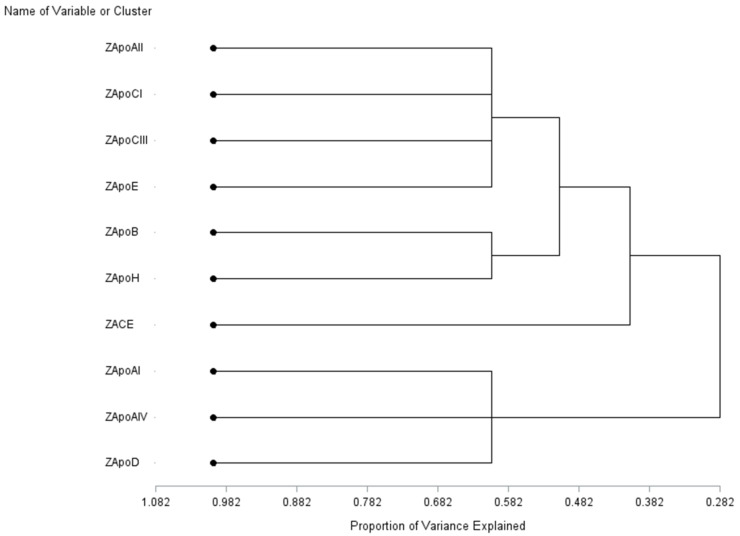
Variable cluster analysis of ten proteins.

Pearson and Spearman correlation coefficients for 10 proteins are presented in Table 2. A total of 10 proteins were clustered into four clusters (Figure 3): ApoAII, ApoCI, ApoCIII, and ApoE (cluster 1), ApoB and ApoH (cluster 2), ACE1 (cluster 3), and ApoAI, ApoAIV and ApoD (cluster 4). Four clusters could explain 60.5% of the total variance. Table 2 displays the strong correlation between variables of ten proteins within clusters.

### 3.3. Comparison of Means Using Independent Samples t Test

Initial analysis was performed to compare means between two groups of each of the 10 proteins using independent *t* test without adjusting for potential covariates. Results are shown in Table 3. There were significant differences between AD and CN in several biomarkers, including ACE1, ApoAII, ApoB, ApoCIII, ApoE and ApoH (*p* values < 0.05, Table 3). Similarly, there were significant differences between MCI and CN in several biomarkers, namely, ACE1, ApoAII, ApoAIV, ApoCI, ApoCIII, ApoD and ApoE. Between CVD and non-CVD, significant differences were observed in four biomarkers, namely, ACE1, ApoAI, ApoB, and ApoH. Additionally, there were significant differences in ApoAII and ApoD between EMD and non-EMD. Moreover, statistically significant differences were found between *ApoE* ε4 allele(s) in ApoB and ApoE proteins.

### 3.4. Analysis Using Multivariable General Linear Model (GLM)

To adjust for potential covariates, multivariable GLM was used. Table 4 shows the results. AD, MCI, CVD and EMD maintained significant associations with ACE1 protein (*p* = 0.0093, 0.0091, < 0.0001 and 0.0265, respectively). AD, CVD and *ApoE* ε4 allele showed significant associations with ApoB protein (*p* = 0.0448, 0.0233 and 0.0255, respectively); while AD and CVD showed associations with ApoH protein (*p* = 0.0031 and 0.0107, respectively). Furthermore, AD, MCI and EMD revealed significant associations with ApoAII protein (*p* =<0.0001, <0.0001 and 0.0193, respectively); while AD, MCI, EMD and *ApoE* ε4 allele showed strong associations with ApoE protein (*p* = 0.0306, < 0.0001, 0.0115 and < 0.0001, respectively).

## 4. Discussion

In this study, we examined possible associations of nine APO proteins and one enzyme with three common health conditions and *ApoE* ε4 genotype. The main findings of this study include: (1) ACE1 and several APO proteins are associated with AD, CVD and EMD, respectively. To our knowledge, this is the first study of the potential association of the 10 biomarkers (9 APO proteins, 1 enzyme) with the three identified chronic health conditions (AD, CVD, and EMD). (2) The *ApoE* ε4 allele is associated with AD and MCI, but not directly associated with CVD and EMD. (3) The *ApoE* ε4 allele is positively associated with ApoB level and negatively associated with ApoE level in CSF.

By using a multivariable GLM, we found that ACE1 is associated with the four health conditions (AD, MCI, CVD, and EMD) after controlling for covariates. The observed inverse relationship in the association of ACE with AD and MCI in the current study is consistent with previous studies, including one that shows that ACE2 activity is significantly reduced in AD, when compared with age-matched controls from human brain samples [36]. ACE has been suggested to play a direct role in regulating the degradation of Aβ. An earlier cross-sectional regression analysis concluded that ACE protein level and CSF activity were significantly lower in subjects with AD, therefore strengthening the hypothesis that ACE degrades Aβ [21]. However, elevated ACE1 level was associated with worse processing speed and a working memory of 18 patients with AD [37], which may be due to differences in sample size (e.g., a small patient population), study design, and different ACE levels (such as ACE 1 or 2). Previous findings suggest that ACE could degrade the B-amyloid. For example, the B-amyloid is one of the pathological markers for AD. The activity of ACE would depend primarily on either the insertion or deletion polymorphism of the *ACE* gene. The use of ACE inhibitors could slow down the progression and deterioration of cognitive functions in affected individuals [38]. In terms of the association of ACE with CVD, a study of soluble ACE2 showed the positive association of this biomarker with myocardial injury and neurohormonal activation, impaired diastolic function, and CVD [39]. A recent study suggests that higher soluble ACE2 levels were associated with significantly higher biomarkers of cardiac injury [39], which supports our current findings. In terms of ACE associated with metabolic-related disorders, a previous study on 90 patients with diabetes reported that urine ACE2 might potentially function as a marker for monitoring the metabolic status [40], which partially supports the current findings. However, the phenotypes of EMD in the current study are broader (including both endocrine diseases and metabolic diseases) and with a large sample size (total of 198 patients with EMD and 320 non-EMD). To the best of our knowledge, this is the first report of a positive association of ACE1 enzyme level with EMD.

Accumulated evidence, including the current findings, suggest that the *ApoE* ε4 allele is associated with AD [41]. However, there has been only a limited study of ApoE protein level in association with diseases, such as AD [42,43]. The results of this current study provide additional evidence: significant inverse associations of ApoE protein levels with AD, MCI, and EMD, as well as *ApoE* ε4 allele. With respect to AD, an inverse association of ApoE protein with AD in the current study is consistent with previous reports in postmortem brain samples [44]. In addition, it has been well established that the ApoE protein modulates the formation of amyloid plaques and neurofibrillary tangles, and that various ApoE isoforms enhance or mitigate AD onset by clear and precise molecular mechanisms [42]. A recent study showed that human CSF from *ApoE* ε4/ε4 carriers had a greater percentage of aggregated ApoE protein compared with CSF from *ApoE* ε3/ε3 carriers [45]. The results of our current study and previous reports, including the accumulation of β-amyloid (Aβ) plaques [45], suggest that ApoE protein plays a central role in AD pathology.

With respect to the association of *ApoE* ε4 allele and ApoE protein with CVD, the results of the current study suggest that CVD showed borderline significant association with ApoE protein (*p* = 0.0675, Table 3), but no association with the *ApoE* ε4 allele. The results of a phenome-wide association study (PheWAS) suggest the association of *ApoE* genotypes with disease risks and found consistent evidence of the *ApoE* ε4 allele and an elevated risk of multiple CVD [2]. A second cross-sectional study with a total of 924 participants concluded that the population carrying the *ApoE* ε4 allele had an increased risk of CVD and type 2 diabetes (T2DM); additionally, these subjects manifested a higher level of lipid profiles [46]. However, the results of our current study did not support *ApoE* ε4 allele association with CVD, which may be due to different sample sizes, study designs, and other factors. Due to its distinct role in lipid metabolism, *ApoE* variants naturally influence cholesterol transport and homeostasis. A recent study suggested that the relation between *ApoE* ε4 and CVD can also determine the risk and prevalence of dementia; however, data have many gaps and have been deemed inconsistent [4]. Recent therapeutic approaches of ApoE protein targeted (from the *ApoE* gene to the ApoE protein and its interactors) have been developed in animal models, which may be ready to be translated to human in the future [47]. The *ApoE* gene is a promising therapeutic target for those conditions, which remains investigated [47]. Accumulated studies demonstrate that lipid metabolism is involved in health conditions, and the ApoE protein, as a major lipid transporter, plays a key role in the pathogenesis of AD [43], CVD, and EMD.

Associations of ApoB, ApoCI, ApoCIII, and ApoH were observed with AD, MCI, CVD and EMD respectively in the current study, including decreased ApoB protein level in CVD, but increased in AD, which are supported by previous studies. For instance, analyses of incident CVD events showed inverse associations with ApoB [48]. Another study indirectly suggests that ApoB is associated with AD, based on their observation that all participants with *ApoE* ε3/ε4 or ε4/ε4 alleles had high levels of ApoB [49]. However, several studies showed an increase in ApoB in CVD including one study, which suggested that elevated ApoB serum levels strongly predict early cardiovascular events [50]. Increasing evidence suggests that ApoB is known to be a more powerful predictor of CVD than conventional lipids [51,52]. A recent study also suggests that ApoB particles drive the atherosclerotic process that leads to the precursors of clinical events [53].

A considerable amount of data have highlighted that a high level of ApoCIII leads to a high cholesterol level, which may influence CVD risk; however, the results of the current study just showed borderline significant association (*p* = 0.0594, Table 4). The findings of inverse association of ApoCIII with AD are supported by a previous study of patients with AD [54]. The results of current and previous studies suggest that individuals with low plasma levels of ApoCIII are at risk for AD. In addition, ApoAII (but not ApoAI) was significantly downregulated, and ApoAIV was upregulated in AD and MCI in the current study. From a study of different ApoA proteins, a recent meta-analysis based on 17 case-control-, two cohort-, and three combined case-control studies (a total of 207 AD and mild cognitive) showed that ApoA-I decreased in AD, while ApoAIV increased [55], which partially supports the current findings. However, this study did not include ApoAII. ApoAIV was found to be upregulated in AD and MCI in our current study. More research is needed to validate the finding, since understanding how changes in cellular cholesterol levels and apolipoprotein homeostasis affect the central nervous system will offer promising novel avenues for the future treatment of neurological disorders [56], such as AD. In addition, there has been limited study of ApoH in association with diseases. A genetic study suggested that *ApoH* polymorphism was associated with some serum lipid parameters in the two ethnic groups, and that rs1801690 near the *ApoH* gene might have racial/ethnic and/or gender-specificity [57]. The ApoH acts as a multi-functional glycoprotein in which it has been correlated with negative health outcomes due to their high heritability [58]. The ApoH, as a circulating protein, is bound to the lipoproteins and contributes to atherosclerotic pathways through its immunological response. One study showed that novel CSF proteins were found to have effects on the inferior and middle temporal cortex, including ApoH in 90 healthy elders [59]. Future validation studies to establish reliable AD biomarkers are needed.

With respect to shared biomarkers among the four conditions (AD, MCI, CVD and EMD), our study showed a strong disease association with *ApoE* ε4 allele and ApoE proteins. AD and CVD are moderately associated with ApoH and MCI, while CVD has an inverse association with ApoD. These are new results uncovered by this work. However, *ApoE* ε4 allele and certain clinical phenotypes have been repeatedly shown to be shared by AD and CVD [10,16]. Its exact mechanism is not yet known, but ApoE binds preferentially to triglyceride-rich, very low density lipoproteins, leading to the downregulation of LDL receptors, which may be involved in CVD development [12]. Previous studies also suggest that structure correctors are considered as a potential therapeutic approach to reduce *ApoE* ε4 pathology in both CVD and neurological disorders, including AD [12].

Several factors may result in discrepancies among the findings from these studies: (1) different ethnicities, as this demographic factor could influence protein levels [55], (2) different types of ACE enzymes: for example, reverse association of ACE1 level with AD in the current study; high expression levels of ACE2 observed in the brain of AD patients [60], (3) different collection tubes for plasma and serum could also contribute to the variation in protein identification by mass spectrometry [61,62], (4) dissimilar protein profiles could be due differences in the sampling procedures [62] and reagents, (5) different stages of AD [55], CVD and EMD among different studies.

We are also aware of strengths and limitations of the present study. Strengths include (1) the comparatively moderate sample size with 109 AD, 356 MCI, 373 CVD, and 198 EMD: (2) identification of shared *ApoE* ε4 allele among AD, MCI, CVD, and EMD. To our knowledge, this is the first study on shared disease-associated allele, levels of ACE1 and nine APO proteins in association with these health conditions. Limitations of the study include (1) some patients with AD and MCI, may also suffer from other health conditions (e.g., CVD and/or EMD); (2) phenotypic heterogeneity with high variation could result in the inconclusive accentuation of biomarkers (*ApoE* ε4 allele, ACE1, and APO proteins). In the future, we will focus on more specific phenotypes, for instance, by using novel methods of the ADNI-based analysis of imaging data, such as MRIs, or of a fusion of information from both MRIs and proteomic data; (3) although we started with a moderate sample size, after further dividing to *ApoE* ε4 1+ and 0, the sample sizes could become comparatively small and, as a result, may not be adequately powered to detect genetic variants exerting smaller effects. Therefore, future large-scale studies with meta-analysis studies are needed to validate the current findings.

## 5. Conclusions

Altogether, we have examined ten protein levels in association with AD, MCI, CVD, EMD, and *ApoE* ε4 allele. To the best of our knowledge, this is the first report of these protein levels (one ACE (namely, ACE1), and nine APOs (namely, ApoAI, ApoAII, ApoAIV, ApoB, ApoCI, ApoCIII, ApoD, ApoE, and ApoH) as biomarkers, either upregulated or downregulated for AD, CVD, and EMD, respectively. We also showed specifically shared *ApoE* ε4 allele and ApoE protein among these health conditions. In addition, shared ApoB and ApoH biomarkers in AD and CVD were identified. Further integrating the current findings could be useful for validation and/or confirmation studies to establish reliable disease biomarkers.

## Figures and Tables

**Table 1:** Descriptive statistics.

Variable	AD vs. MCI vs. CN Mean ± SD or Count	*F*/*χ*^2^, *p*	CVD vs. Non-CVD Mean ± SD or Count	*t*/*χ*2, *p*	EMD vs. Non-EMD Mean ± SD or Count	*t*/*χ*^2^, *p*
*N*	109 vs. 356 vs.53		373 vs. 145		198 vs. 320	
Age	74.9 ± 8.0	0.09, 0.9176	75.4 ± 7.2 74.3 ± 7.7	1.60, 0.1099	75.5 ± 6.9 74.8 ± 7.6	1.08, 0.2820
	75.1 ± 7.3					
	75.4 ± 5.7					
Gender						
Male	64 vs. 232 vs. 28	3.86, 0.1449	244 vs. 80	4.68, 0.0306	115 vs. 209	2.73, 0.0984
Female	45 vs. 124 vs. 25		129 vs. 65		83 vs. 111	
Edu	15.2 ± 3.1	1.55, 0.2134	15.5 ± 3.0 15.9 ± 2.8	−1.17, 0.2407	15.5 ± 3.0 15.7 ± 2.9	−0.82, 0.4141
	15.7 ± 3.0					
	15.9 ± 2.7					
*ApoE* ε4 allele						
0	36 vs. 164 vs. 49	52.28, <0.0001	177 vs. 72	0.20, 0.6524	98 vs. 151	0.26, 0.6095
1+	73 vs. 192 vs. 4		196 vs. 73		100 vs. 169	

Abbreviations: AD: Alzheimer disease; CN: Cognitive normal; MCI: Mild cognitive impairment; CVD: Cardiovascular disease, non-CVD; EMD: Endocrine-metabolic diseases, non-EMD; SD: Standard deviation. *p* value is based on Chi-square test or F test or t test in ANOVA. Due to missing values, the total number for some variables was less than 518.

**Table 2:** Correlation analysis of ten proteins.

Variable	ACE1	ApoAI	ApoAII	ApoAIV	ApoB	ApoCI	ApoCIII	ApoD	ApoE	ApoH
ACE1	1	−0.0567 0.1976	0.0818 0.0629	0.1056 0.0162	0.0039 0.9291	−0.0381 0.3870	0.0914 0.0377	−0.0021 0.9626	0.0375 0.3949	0.0235 0.5936
ApoAI	−0.0515 0.2418	1	0.3045 <0.0001	0.1378 0.0017	−0.0941 0.0323	0.4660 <0.0001	0.1845 <0.0001	0.3811 <0.0001	0.1096 0.0126	0.0150 0.7328
ApoAII	0.1004 0.0222	0.3062 <0.0001	1	0.0094 0.8302	0.1424 0.0012	0.5758 <0.0001	0.4626 <0.0001	−0.0683 0.1204	0.2354 <0.0001	0.2035 <0.0001
ApoAIV	0.1055 0.0163	0.1612 0.0002	−0.0139 0.7562	1	0.0410 0.3518	−0.0142 0.7471	0.0446 0.3112	0.1037 0.0182	−0.0119 0.7870	0.0752 0.0874
ApoB	−0.0223 0.6128	−0.1117 0.0109	0.1317 0.0027	0.0198 0.6536	1	0.2989 <0.0001	0.2126 <0.0001	0.0156 0.7227	0.2269 <0.0001	0.2044 <0.0001
ApoCI	−0.0199 0.6519	0.4548 <0.0001	0.5698 <0.0001	−0.0294 0.5037	0.2815 <0.0001	1	0.5908 <0.0001	0.1934 <0.0001	0.4515 <0.0001	0.2111 <0.0001
ApoCIII	0.0930 0.0343	0.1845 <0.0001	0.4820 <0.0001	0.0089 0.8396	0.1951 <0.0001	0.6041 <0.0001	1	−0.0242 0.5831	0.4438 <0.0001	0.2170 <0.0001
ApoD	0.0021 0.9620	0.3740 <0.0001	−0.0799 0.0689	0.1270 0.0038	−0.0204 0.6430	0.1817 <0.0001	−0.0216 0.6242	1	0.1151 0.0087	0.0756 0.0857
ApoE	0.0381 0.3867	0.1077 0.0142	0.2637 <0.0001	−0.0425 0.3340	0.2303 <0.0001	0.4518 <0.0001	0.4503 <0.0001	0.1305 0.0029	1	0.1419 0.0012
ApoH	0.0481 0.2744	−0.0047 0.9154	0.2453 <0.0001	0.0753 0.0869	0.1743 <0.0001	0.1998 <0.0001	0.2529 <0.0001	0.0038 0.9320	0.1366 0.0018	1

Above the diagonal is the Pearson correlation coefficient; below the diagonal is Spearman correlation coefficient. Lower value in each cell denotes the *p*-value.

**Table 3:** Comparison of means using independent samples *t*-test.

Variable	AD vs. CN *t*, *p*	MCI vs. CN *t*, *p*	CVD vs. Non-CVD *t*, *p*	EMD vs. Non-EMD *t*, *p*	*ApoE ε4-1+ vs 0* *t*, *p*
ACE1	−2.64, 0.0085	−2.67, 0.0077	4.59, <0.0001	2.39, 0.0174	−0.72, 0.4724
ApoAI	0.84, 0.4030	−0.57, 0.5681	−2.22, 0.0266	−0.65, 0.5186	−1.55, 0.1219
ApoAII	−4.43, <0.0001	−6.17, <0.0001	0.42, 0.6752	2.43, 0.0156	−1.84, 0.0661
ApoAIV	1.64, 0.1016	3.60, 0.0004	0.48,0.6344	0.08, 0.9345	−1.09, 0.2755
ApoB	2.95, 0.0033	1.03, 0.3025	−2.37, 0.0182	−0.74, 0.4607	3.20, 0.0015
ApoCI	0.94, 0.3458	−2.90, 0.0039	−1.77, 0.0733	−0.03, 0.9776	−0.83, 0.4057
ApoCIII	−2.76, 0.0059	−2.46, 0.0141	1.48, 0.1392	1.56, 0.1190	−1.45, 0.1475
ApoD	0.37, 0.7113	−2.82, 0.0050	−1.42, 0.1564	−2.50, 0.0126	−1.55, 0.1226
ApoE	−5.05, <0.0001	−6.88, <0.0001	−1.83. 0.0675	−1.76, 0.0794	−10.73, <0.0001
ApoH	3.05, 0.0024	−0.49, 0.6222	2.99, 0.0029	1.56, 0.1191	0.05, 0.9580

Abbreviations: AD: Alzheimer disease; CN: Cognitive normal; MCI: Mild cognitive impairment; CVD: Cardiovascular disease; EMD: Endocrine-metabolic diseases; ACE1: Angiotensin-converting enzyme 1. *p* value is based on independent samples *t* test.

**Table 4:** Multivariable GLM for ten proteins.

Variable	AD vs. CN *t*, *p*	MCI vs. CN *t, p*	CVD vs. Non-CVD *t*, *p*	EMD vs. Non-EMD *t*, *p*	*ApoE ε*4-1+ vs. 0 *t*, *p*
ACE1	−2.61, 0.0093	−2.62, 0.0091	4.40, <0.0001	2.22, 0.0265	0.02, 0.0.9863
ApoAI	191, 0.0573	0.66, 0.5089	−1.20, 0.2319	−1.37,0.1728	−1.65, 0.1004
ApoAII	−4.12, <0.0001	−5.66, <0.0001	0.82, 0.4127	2.35, 0.0193	−0.50, 0.6166
ApoAIV	2.30, 0.0219	4.12, <0.0001	0.83, 0.4054	0.01, 0.9975	−2.00, 0.0455
ApoB	2.01, 0.0448	0.32, 0.7458	−2.28, 0.0233	−0.62, 0.5340	2.24, 0.0255
ApoCI	1.60, 0.1011	−2.07, 0.0392	−0.82, 0.3917	−0.55, 0.5797	−1.07, 0.2863
ApoCIII	−2.42, 0.0159	−1.88, 0.0608	1.89, 0.0594	1.11, 0.2686	−0.57, 0.5696
ApoD	0.86, 0.3904	−2.51, 0.0124	−1.60, 0.1102	−2.43, 0.0155	−1.32, 0.1860
ApoE	−2.17, 0.0306	−4.31, <0.0001	−1.50. 0.1333	−2.54, 0.0115	−9.37, <0.0001
ApoH	2.98, 0.0031	−0.45, 0.6494	2.56, 0.0107	1.28, 0.1995	−0.63, 0.5280

Abbreviations: Alzheimer disease; CN: Cognitive normal; MCI: Mild cognitive impairment; CVD: Cardiovascular disease; EMD: endocrine-metabolic diseases; *p* value is based on *t* test in multivariable GLM.

## Data Availability

Data used in the preparation of this article were obtained from the Alzheimer’s Disease Neuroimaging Initiative (ADNI) database (adni.loni.usc.edu).
